# Stalking the Fourth Domain in Metagenomic Data: Searching for, Discovering, and Interpreting Novel, Deep Branches in Marker Gene Phylogenetic Trees

**DOI:** 10.1371/journal.pone.0018011

**Published:** 2011-03-18

**Authors:** Dongying Wu, Martin Wu, Aaron Halpern, Douglas B. Rusch, Shibu Yooseph, Marvin Frazier, J. Craig Venter, Jonathan A. Eisen

**Affiliations:** 1 Department of Evolution and Ecology, Department of Medical Microbiology and Immunology, University of California Davis Genome Center, University of California Davis, Davis, California, United States of America; 2 The J. Craig Venter Institute, Rockville, Maryland, United States of America; 3 The J. Craig Venter Institute, La Jolla, California, United States of America; 4 University of Virginia, Charlottesville, Virginia, United States of America; Smithsonian Institution National Zoological Park, United States of America

## Abstract

**Background:**

Most of our knowledge about the ancient evolutionary history of organisms has been derived from data associated with specific known organisms (i.e., organisms that we can study directly such as plants, metazoans, and culturable microbes). Recently, however, a new source of data for such studies has arrived: DNA sequence data generated directly from environmental samples. Such metagenomic data has enormous potential in a variety of areas including, as we argue here, in studies of very early events in the evolution of gene families and of species.

**Methodology/Principal Findings:**

We designed and implemented new methods for analyzing metagenomic data and used them to search the Global Ocean Sampling (GOS) Expedition data set for novel lineages in three gene families commonly used in phylogenetic studies of known and unknown organisms: small subunit rRNA and the *recA* and *rpoB* superfamilies. Though the methods available could not accurately identify very deeply branched ss-rRNAs (largely due to difficulties in making robust sequence alignments for novel rRNA fragments), our analysis revealed the existence of multiple novel branches in the *recA* and *rpoB* gene families. Analysis of available sequence data likely from the same genomes as these novel *recA* and *rpoB* homologs was then used to further characterize the possible organismal source of the novel sequences.

**Conclusions/Significance:**

Of the novel *recA* and *rpoB* homologs identified in the metagenomic data, some likely come from uncharacterized viruses while others may represent ancient paralogs not yet seen in any cultured organism. A third possibility is that some come from novel cellular lineages that are only distantly related to any organisms for which sequence data is currently available. If there exist any major, but so-far-undiscovered, deeply branching lineages in the tree of life, we suggest that methods such as those described herein currently offer the best way to search for them.

## Introduction

During the last 30 years, technological advances in nucleic acid sequencing have led to revolutionary changes in our perception of the evolutionary relationships among all species as visualized in the *tree of life*. The first revolution was spawned by the work of Carl Woese and colleagues who, through sequencing and phylogenetic analysis of fragments of rRNA molecules, demonstrated how the diverse kinds of known cellular organisms could be placed on a single tree of life [1], [2], [3]. Most significantly, their analyses revealed the existence of a third major branch on the tree; the Archaea (then referred to as Archaebacteria) took their place along with the Bacteria and the Eukaryota [2]. Several factors make rRNA genes exceptionally powerful for this purpose, the most important being perhaps that highly conserved, homologous rRNA genes are present in all cellular lineages. To this day, analyses of rRNA genes continue to clarify and extend our knowledge of the evolutionary relationships among all life forms [4], [5].

For microbial organisms, this approach was restricted to the minority that could be grown in pure culture in the laboratory until Norm Pace and colleagues showed that one could sequence rRNAs directly from environmental samples [6], [7]. Initially, the methodology was cumbersome. However, this changed with the development of the polymerase chain reaction (PCR) methodology [8]. PCR generates many copies of a target segment of DNA, which in turn facilitates cloning and sequencing of that segment. However, delineation of the segment to be amplified requires *primers*, i.e., short segments of DNA whose nucleotide sequence is complementary to the DNA flanking the target. Because rRNA genes contain regions that are very highly conserved, “universal primers” can be used for PCR amplification of those genes even in environmental samples [9], [10]. Thus, in principle, one can use PCR to amplify the rRNA genes from all organisms in a sample in a culture-independent manner.

PCR-based studies have now characterized microbes from diverse habitats and have provided many fundamental new insights into microbial diversity. For example, we now realize that, in most environments, the culturable microbes represent but a small fraction of those present. Furthermore, phylogenetic analysis of the rRNA genes thus found enables one to assign those sequences to groups within the bacterial, archaeal, or eukaryotic domains of life (or to viral groups), a process known as *phylotyping*. This has revealed the presence of dozens of major, but previously undiscovered, lineages that have no cultured members [5]. With the development of considerably improved sequencing technologies, rRNA PCR surveys have become a routine tool for characterization of microbial communities.

Although rRNA PCR studies have provided a major foundation for today's environmental microbiology, this approach is not without its limitations. Notably, the “universal” primers are not truly universal. Even the best-designed ones fail to amplify the targeted genes in some lineages while preferentially amplifying those in others [11]. Furthermore, phylogenetic trees based on rRNA sequences may not accurately reflect the evolutionary history of the source organisms due to the occurrence of lateral gene transfer, different rates of evolution in different lineages, or similarities produced by the convergent evolution of rRNA sequences from distantly related species [12], [13], [14], [15]. Generating alignments of rRNA genes can sometimes be challenging. Furthermore, because the copy number of rRNA genes varies in different species [16], [17], the number of sequences observed in an environment cannot be used to directly infer the number of cells of any particular type [18] [19]. For these and other reasons, it is generally considered to be important to combine conclusions derived from rRNA sequence analysis with other types of information (e.g., microscopy, analysis of other macromolecules, etc). In terms of sequence information, this would mean generating data for other genes. This can be readily achieved for culturable organisms; phylogenetic analysis of protein coding genes, and even *phylogenomic* analysis of whole genomes, has become a standard procedure [20] [21], [22]. But unfortunately, despite considerable effort, no one has developed a robust PCR-based method for cloning and sequencing protein-coding genes from unknown uncultured organisms. Note – if you know reasonably detailed information about the taxonomy of the targeted uncultured organisms, one can get PCR of protein coding genes to work reasonably well. A major inherent obstacle in PCR of protein coding genes from unknown organisms is the degenerate nature of the genetic code. Even if the amino acid sequence of a highly conserved protein domain were identical across species, the primers for PCR amplification would have to be degenerate. Thus, although PCR surveys of protein-coding genes have revealed interesting findings, they are clearly limited somewhat in scope (e.g., [23]).

Due to these factors, the community has faced a bit of a quandary regarding the characterization of uncultured organisms. Although rRNA analysis is extraordinarily powerful, the window it provides into the microbial world is clearly imperfect. It is possible that additional major branches in the tree of life might exist, branches that have been missed due to the limitations of rRNA PCR. To resolve this required ways to clone rRNA genes without the biases introduced by PCR, as well as unbiased methods for obtaining data on other genes from uncultured species. Fortunately, both are now provided by metagenomic analysis. *Metagenomics*, broadly defined, is the sequencing of portions of the genomes of all organisms present in an environmental sample [24] [25]. It generates sequence data not only for rRNA genes, but for all sequences from the genomes of all organisms present, in a relatively unbiased manner (or at least with a different bias than that inherent in PCR) [26].

The application of metagenomic analysis has accelerated the rapid rate of advancement in the study of uncultured microbes that began with the advent of rRNA analysis (e.g., [19], [27], [28], [29], [30], [31]). Metagenomics has now enabled the phylogenetic characterization of many entire communities. For example, our analysis of the Sargasso Sea metagenomic data effectively used both protein-coding and rRNA sequences for phylotyping, in much the same way as had been done with rRNA PCR data [19]. Furthermore, by including protein-coding genes, metagenomics can more accurately predict the biology of the organisms sampled, thus disclosing not only who is out there, but also what they are doing [32], [33], [34].

Previous usage of metagenomic data for phylogenetic typing of organisms focused primarily on assigning metagenomic sequences to specific known groups of organisms (e.g., see [22]). Here we report our exploration of the potential use of metagenomic data to answer a simpler, but perhaps more fundamental, question: *Can we identify novel rRNAs or protein-coding genes that suggest the existence of additional major branches on the tree of life?* The answer, surprisingly, is yes. We present here our findings, along with some likely explanations—including the possibility that there are indeed other major branches on the tree of life yet to be characterized.

## Results and Discussion

**Note:** Much of the analysis reported in this paper was initially done during 2004–2007 using the data sets available at that time. Some subsequent follow-up analyses included datasets released since 2007 but not all currently available datasets were analyzed.

### Searching for novel branches in the rRNA tree

We sought here to address a single question: *Are there small-subunit rRNAs (ss-rRNAs) encoded by this metagenomic data set that represent novel lineages that branch closer to the base of the tree of life than any known ss-rRNAs?*

Since the largest metagenomic data sets available when this work was begun came from the Sorcerer II Global Ocean Sampling Expedition (GOS) [35], [36], we focused on the GOS data. We realized that there were too many ss-rRNA genes in this data set for manual analysis, and the automated methods available at that time were designed to assign rRNA genes to known phylogenetic groups, *not* to detect novel rRNA genes [37], [38]. Therefore, we developed an automated screening system (STAP) for detecting ss-rRNA genes that branch very deeply in the tree of life (see Methods). In summary, this automated system: (1) identifies ss-rRNA coding sequences in the metagenomic data set; (2) generates an alignment of each of those ss-rRNA gene sequences against a prealigned set of representative ss-rRNA sequences from the three domains of life; (3) builds phylogenetic trees from each of these alignments; and (4) identifies those trees in which the environmental sequence branches very deeply, i.e., either between the three domains or as one of the deepest branches within a domain (assuming that each domain is a monophyletic group) [39].

Using this approach, we examined the entire GOS data set of 14,689 putative ss-rRNA sequences and identified 18 sequences that met our multiple criteria for potentially being deep branching (JCVI reads: 1098241, 1092963341190, 1091140405652, 318, 1105333456790, 1103242712700, 1105499913772, 1103242587147, 1108829508267, 1092959443067, 1092405960359, 1092402545613, 1093018267888, 1095522122248, 1093018199876, 1092351161318, 1092381601933, 1095527007809). Most importantly, these 18 could not be assigned to any of the three domains by STAP and they are each positioned near a domain separation point in a maximum likelihood tree that includes representatives from all three domains. However, more detailed examination of those alignments and trees disclosed problems in the alignments for all of the candidate novel sequences. Some alignments were of low quality due to the insertion of too many gaps in the novel sequence. Alignment quality is critical to phylogenetic analysis because the alignment is a hypothesis concerning the homology (common ancestry) of the residues at the same position in each of the aligned sequences (*positional homology*). A tree built from a flawed alignment may not reflect actual evolutionary relationships. Additionally, many of the novel rRNA gene sequences aligned well only for very short regions (<300 bp). It seems plausible that none of these novel sequences are actual ss-rRNA genes.

These difficulties served to confirm that the methods available (or at least the ones we were using for this high throughput approach) were not robust enough to identify novel ss-rRNA genes in an automated manner. Most of our problems were inherent in attempting to generate high quality alignments of short sequences that are only distantly related to known ss-rRNA genes. Furthermore, alignment of novel rRNA sequences can be challenging because often it is the secondary and tertiary structure of the molecule, rather than the primary sequence, that is highly conserved. Our attempts to improve the alignments based on *de novo* prediction of folding for the novel ss-rRNAs also fell short, likely because most of the novel ss-rRNA sequences were fragments and the folding algorithms work best on complete sequences.

Even when one has high quality rRNA gene sequence alignments, phylogenetic analysis involving very deep branches in the tree of life can still be difficult due to inherent complications, such as convergent evolution due to GC content effects [40], [41], [42]. Thus if there exist phylogenetically very deeply branching rRNA genes in metagenomic data sets, our methods were not ideally suited for finding them. (We note that since we conducted this original analysis, new alignment methods for rRNA genes have been developed that may eventually help to resolve some of these issues. [43])

### Analysis of the RecA superfamily

Due to the difficulties discussed above, we turned to protein-coding genes in our search for novel branches in the tree of life. To take the place of the ss-rRNA genes, we needed a protein-coding gene that was both universal and widely studied. For our initial test we chose the *recA* superfamily that includes the genes encoding a diversity of recombinases that effect homologous recombination of DNA (e.g., RecA in Bacteria, RadA and RadB in Archaea, Rad51, Rad57 in Eukaryota and UvsX in phage). This is a nearly universal gene family; homologs are found in almost all species of Bacteria, Archaea, and Eukaryotes, and even in some viruses. The few bacterial groups that lack homologs have greatly reduced genomes (e.g., some endosymbionts [44], [45], some mycoplasmas [46]). In addition, it is one of the most widely sequenced protein-coding gene families due to its importance in recombination, DNA repair, and sex, and because of its usefulness as a phylogenetic marker in studies of the Bacteria [19], [47], [48], Archaea [23], and Eukaryota [49] [50].

Our question became: *Does the GOS metagenomic data set encode any new classes of proteins within the RecA superfamily?* To answer this, our first step was to retrieve representative sequences encoding known subfamilies of the RecA superfamily including the bacterial RecA, the archaeal RadA and RadB, the viral UvsX, and the eukaryotic DMC1, Rad51, Rad51B, Rad51C, and Rad51D subfamilies. The gene sequences of representatives of these known RecA superfamilies, as well as other RecA homologs, were then used to query the predicted proteins from the GOS metagenomic environmental data to identify homologs. In addition, we used HMM searches to identify as many RecA superfamily members as possible in Genbank and complete genome data sets.

In total, 4677 RecA superfamily members were identified from published microbial genomes [51], the NRAA database, and the GOS metagenomic data set (4125 of these were from the GOS data). At the time this analysis was done, our assessment was that there were no reliable methods capable of robustly both aligning and inferring phylogenetic trees for this many sequences. Therefore, we used an alternative approach by first partitioning the superfamily into subgroups. This partitioning was carried out at the protein level using a Lek clustering method (see Methods) previously employed for protein superfamily classification [52]. In essence, the goal here was to first subdivide the superfamily into subgroups – and to then select representatives of each subgroup for further analysis. Such “pre-clustering” is a common approach to analysis of large protein superfamilies though this is perhaps the first use of the Lek method for these purposes.

**Figure 1:**
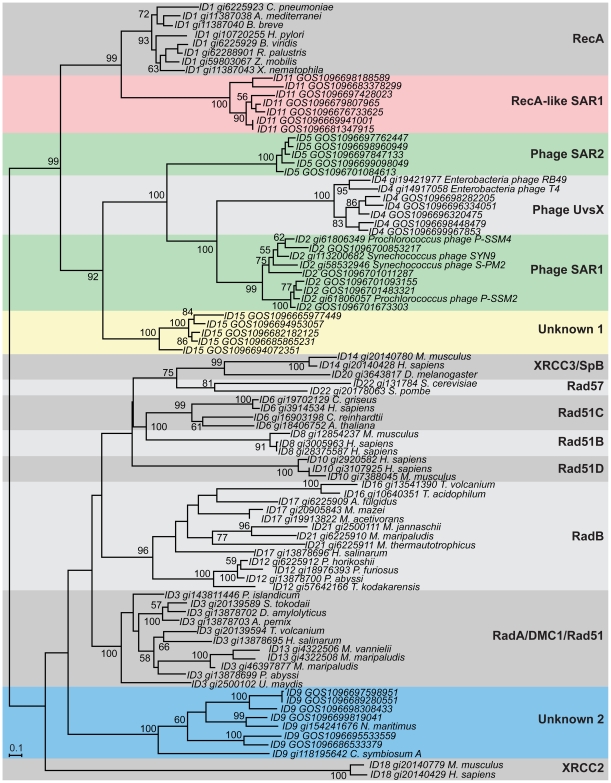
Phylogenetic tree of the RecA superfamily. All RecA sequences were grouped into clusters using the Lek algorithm. Representatives of each cluster that contained >2 members were then selected and aligned using MUSCLE. A phylogenetic tree was built by from this alignment using PHYML; bootstrap values are based on 100 replicas. The Lek cluster ID precedes each sequence accession ID. Proposed subfamilies in the RecA superfamily are shaded and given a name on the right. Five of the proposed subfamilies contained only GOS sequences at the time of our initial analysis (RecA-like SAR, Phage SAR1, Phage SAR2, Unknown 1 and Unknown 2) and are highlighted by colored shading. As noted on the tree and in the text, sequences from two Archaea that were released after our initial analysis group in the **Unknown 2 subfamily.**

Using this method, 23 clusters containing more than two protein sequences were identified (Table 1). A few of these clusters included only short fragmentary peptides. Though these clusters might truly include phylogenetically novel sequences, we considered it likely that their apparent novelty was an artifact of the fragmentary nature of these peptides. Thus these were omitted from further analysis. Of the remaining clusters it was important to determine whether each corresponded to a phylogenetic group (since some clustering methods can produce groupings that are not consistent with phylogenetic relationships). To investigate this, we selected representative samples from each Lek-generated cluster and then built phylogenetic trees for these representatives (Figure 1). Overall, comparison of the trees with the Lek-generated clusters indicates that the Lek clustering reflects phylogenetic relationships. Specifically, the clusters are almost perfectly congruent with the tree. Within the tree, each cluster appears to be a monophyletic grouping, thus demonstrating that the clustering algorithm is robust at this level. In addition, our clusters are virtually identical to the groupings identified by Lin *et al.* [53], with the exception of new clades in our results that contain only GOS metagenome sequences (they did not analyze metagenomic data) (see below).

**Table 1:** RecA superfamily clusters.

Cluster ID	Corresponding Subfamily (see Figure 1)	Corresponding Group in Lin *et al.* [53]	Comments	GOS Only	Number of GOS Sequences
1	RecA	RecA			2830
11	RecA-like SAR1	n/a	Novel	+	10
5	Phage SAR2	n/a	Novel	+	68
4	Phage UvsX	n/a			73
2	Phage SAR1	n/a	Found in cyanophage by subsequent sequencing	+	824
15	Unknown 1		Novel	+	6
14	XRCC3/SpB	Radb-XRCC3			0
20	XRCC3/SpB	Radb-XRCC3			0
22	Rad57	Radb-XRCC2			0
6	Rad51C	Radb-Rad51C			1
8	Rad51B	Radb-Rad51B			2
10	Rad51D	Radb-Rad51D			0
16	RadB	Radb-RadB			0
17	RadB	Radb-RadB			0
21	RadB	Radb-RadB			0
12	RadB	Radb-RadB			0
3	RadA/DMC1/Rad51	Rada			101
13	RadA/DMC1/Rad51	Rada			0
**9	Unknown 2	n/a	Representatives found in Archaea by subsequent sequencing	+	19
18	XRCC2	Radb-XRCC2			0
*7	RecA*	RecA	RecA fragment	+	29
*19	RecA*	RecA	RecA fragment	+	5
*23	RecA*	RecA	RecA fragment	+	3

A Lek protein clustering method was applied to all RecA superfamily members retrieved from the NRAA database, microbial genomes, and the GOS data set. The 23 clusters containing more than two sequences are listed. Clusters that contain only sequences from the GOS data set are noted as “GOS only.” When a cluster can be mapped to a RecA subfamily identified by Lin *et al.* [53], the family designation from that paper is shown in column 3.

*These clusters of RecA fragments from the GOS data set were not included in the phylogenetic tree (Figure 1).

**Although cluster 9 contained only GOS sequences at the time of the initial analysis, it was subsequently found to include marine archaeal homologs from more recent genome sequencing projects.

Based on the clusters and the tree structure, we divided the RecA superfamily into the 15 major grouping labeled in the tree (Figure 1). We refer to these as *subfamilies* of the RecA *superfamily*. Each subfamily contains sequences from one or more of the Lek clusters. At the time of the initial analysis, all sequences in five of these subfamilies (**RecA-like SAR1**, **Phage SAR2**, **Phage SAR1**, **Unknown 1**, and **Unknown 2**) had been found only in environmental metagenomic data. These potentially represented novel previously unknown RecA-related subfamilies. The other 10 groups corresponded to known RecA subfamilies (Table 1). We note, though there is not a perfect one to one mapping of RecA clusters to subfamilies all five of the novel RecA subfamilies included sequences from only one cluster each (**RecA-like SAR1** = cluster 11, **Phage SAR2** = cluster 5, **Phage SAR1** = cluster2, **Unknown 1** = cluster 15, and **Unknown 2** = cluster 9).

What do these novel RecA-related subfamilies and sequences represent? Given their high degree of sequence similarity to proteins in the RecA superfamily, all of which are known to play some role in homologous recombination, it is likely that the members of these new subfamilies are also involved in homologous recombination.

What can we say about the organisms that were the sources of these novel sequences? Two of the five novel subfamilies (**Phage SAR1** and **Phage SAR2**) are reasonably closely related to known phage UvsX proteins (Figure 1) and thus we conclude that the sequences in these groups are likely of phage origin. Analysis of the flanking regions of these sequences indicates that the genes encoding proteins the **Phage SAR1** subfamily are located near protein coding genes that are phage- or virus-related (Table 2). In addition, subsequent sequencing projects carried out after our initial analysis showed that some of the sequences in the Phage SAR1 subfamily are in fact from cyanophages [54], [55].

**Table 2:** Genes linked to sequences in the novel RecA subfamilies.

Subfamily	RecA Accession	Accession of Linked Gene	Assembly ID	Neighboring Gene Description	Taxonomy Assignment
Phage-SAR1	1096700853217	1096700853219	1096627374158	gp43	Viruses/Phages
Phage-SAR1	1096701673303	1096701673301	1096627382978	T4-like DNA polymerase	Viruses/Phages
Phage-SAR1	1096701673303	1096701673305	1096627382978	T4-like DNA primase-helicase	Viruses/Phages
Phage-SAR2	1096697847133	1096697847135	1096627014936	GDP-mannose 4,6-dehydratase	Bacteria
Phage-SAR2	1096697847133	1096697847149	1096627014936	methyltransferase FkbM	Bacteria
Unknown2	1096695533559	1096695533561	1096528150039	ATP-dependent helicase	Archaea
Unknown2	1096698308433	1096698308421	1096627021375	ATP-dependent RNA helicase	Archaea
Unknown2	1096698308433	1096698308423	1096627021375	replication factor A	Archaea
Unknown2	1096698308433	1096698308425	1096627021375	S-adenosylmethionine synthetase	Bacteria
Unknown2	1096698308433	1096698308427	1096627021375	cobalt-precorrin-6A synthase	Archaea
Unknown2	1096698308433	1096698308429	1096627021375	NADH ubiquinone dehydrogenase	Bacteria
Unknown2	1096698308433	1096698308431	1096627021375	CbiG protein	Bacteria
Unknown2	1096698308433	1096698308443	1096627021375	ATP-binding protein of ABC transporter	Bacteria
Unknown2	1096698308433	1096698308435	1096627021375	chaperone protein dnaJ	Eukaryota
Unknown2	1096698308433	1096698308445	1096627021375	small nuclear riboprotein protein snRNP	Archaea
Unknown2	1096699819041	1096699819039	1096627295379	S-adenosylmethionine synthetase	Bacteria
Unknown2	1096699819041	1096699819043	1096627295379	replication factor A	Bacteria
Unknown2	1096699819041	1096699819047	1096627295379	snRNP Sm-like protein	Archaea
Unknown2	1096686533379	1096686533339	1096627390330	ATP-dependent helicase	Archaea
Unknown2	1096686533379	1096686533341	1096627390330	deoxyribodipyrimidine photolyase-related	Bacteria
Unknown2	1096686533379	1096686533343	1096627390330	Glycyl-tRNA synthetase alpha2 dimer	Archaea
Unknown2	1096686533379	1096686533345	1096627390330	RNA-binding protein	Bacteria
Unknown2	1096686533379	1096686533347	1096627390330	cobyrinic acid a,c-diamide synthase	Archaea
Unknown2	1096686533379	1096686533349	1096627390330	sdoxyribodipyrimidine photolyase	Archaea
Unknown2	1096686533379	1096686533351	1096627390330	DNA primase small subunit	Archaea
Unknown2	1096686533379	1096686533353	1096627390330	cobalt-precorrin-6A synthase	Archaea
Unknown2	1096686533379	1096686533355	1096627390330	cobalamin biosynthesis CbiG	Bacteria
Unknown2	1096686533379	1096686533359	1096627390330	DNA primase large subunit	Archaea
Unknown2	1096686533379	1096686533361	1096627390330	aldo/keto reductase	Bacteria
Unknown2	1096686533379	1096686533365	1096627390330	AP endonuclease	Archaea
Unknown2	1096686533379	1096686533369	1096627390330	ATP-dependent helicase	Archaea
Unknown2	1096686533379	1096686533371	1096627390330	translation initiation factor 2 alpha subunit	Archaea
Unknown2	1096686533379	1096686533373	1096627390330	translation initiation factor 2 alpha subunit	Archaea
Unknown2	1096686533379	1096686533375	1096627390330	sirohydrochlorin cobaltochelatase CbiXL	Bacteria
Unknown2	1096686533379	1096686533377	1096627390330	glutamate racemase	Bacteria
Unknown2	1096686533379	1096686533383	1096627390330	glycosyl transferase	Eukaryota
Unknown2	1096686533379	1096686533387	1096627390330	deoxyribodipyrimidine photolyase	Bacteria
Unknown2	1096686533379	1096686533389	1096627390330	AP endonuclease	Archaea
Unknown2	1096686533379	1096686533393	1096627390330	cbiC protein	Archaea
Unknown2	1096686533379	1096686533399	1096627390330	deoxyribodipyrimidine photolyase	Bacteria
Unknown2	1096686533379	1096686533405	1096627390330	cob(I)alamin adenosyltransferase	Bacteria
Unknown2	1096686533379	1096686533407	1096627390330	Phosphohydrolase	Bacteria
Unknown2	1096686533379	1096686533409	1096627390330	glycyl-tRNA synthetase	Archaea
Unknown2	1096686533379	1096686533415	1096627390330	30S ribosomal protein S6	Archaea
Unknown2	1096686533379	1096686533421	1096627390330	nuclease	Archaea
Unknown2	1096686533379	1096686533423	1096627390330	phosphohydrolase	Bacteria
Unknown2	1096686533379	1096686533427	1096627390330	cobalt-precorrin-3 methylase	Archaea
Unknown2	1096686533379	1096686533429	1096627390330	universal stress family protein	Bacteria
Unknown2	1096686533379	1096686533473	1096627390330	aryl-alcohol dehydrogenases related oxidoreductases	Eukaryota
Unknown2	1096686533379	1096686533505	1096627390330	snRNP Sm-like protein Chain A	Eukaryota
Unknown2	1096689280551	1096689280549	1096627650434	S-adenosylmethionine synthetase	Bacteria
RecA-like SAR1	1096683378299	1096683378297	1096627289467	DNA polymerase III alpha subunit	Bacteria
Unknown1	1096694953057	1096694953059	1096520459783	FKBP-type peptidyl-prolyl cis-trans isomerase	Archaea
Unknown1	1096665977449	1096665977451	1096627520210	single-stranded DNA binding protein	Viruses/Phages
Unknown1	1096682182125	1096682182127	1096628394294	DNA polymerase I	Bacteria

Five RecA subfamilies were identified as being novel (i.e., only seen in metagenomic data) in our initial analyses. GOS metagenome assemblies that encode members of these subfamilies were identified and the genes neighboring the novel RecAs were characterized. The neighboring gene descriptions are based on the top BLASTP hits against the NRAA database; taxonomy assignments are based on their closest neighbor in phylogenetic trees built from the top NRAA BLASTP hits.

**Figure 2:**
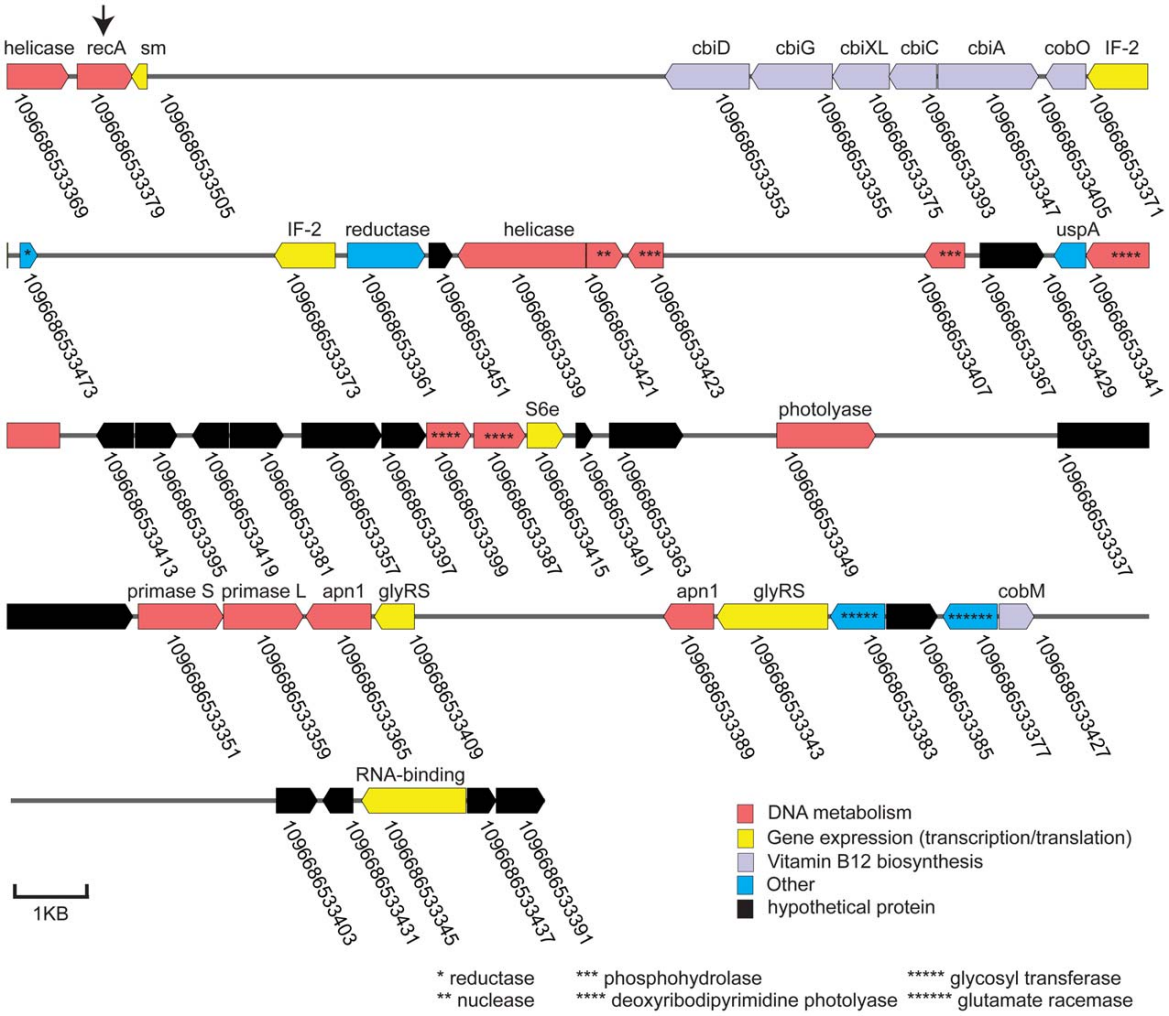
The largest assembly from the GOS data that encodes a novel RecA subfamily member (a representative of subfamily Unknown 2). This GOS assembly (ID 1096627390330) encodes 33 annotated genes plus 16 hypothetical proteins, including several with similarity to known archaeal genes (e.g., DNA primase, translation initiation factor 2, Table 2). The arrow indicates a novel *recA* homolog from the Unknown 2 subfamily (cluster ID 9).

**The Unknown 2 subfamily** is likely of archaeal origin based upon two lines of evidence. First, one of its members was found on a large assembly along with many other protein coding genes, including some that are generally considered to be useful phylogenetic markers (Figure 2). Phylogenetic analysis of all of those genes showed that a majority of them, including the phylogenetic markers, grouped with Archaea (Table 2). Subsequently we found that the RadA-like proteins from the archaeotes *Cenarchaeum symbiosum A* [56] and *Nitrosopumilus maritimus SCM1* (unpublished) also fall within this major group.

**The RecA-like SAR1** subfamily appears be a sister group to the traditional bacterial RecA proteins (Figure 1) and thus we use the prefix “RecA-like” for it. We note though this group is only peripherally related to the bacterial RecAs and is itself quite novel in terms of sequence patterns.

The **Unknown 1** is not particularly closely related to any known groups.

### The RpoB protein superfamily shows qualitatively similar patterns to the RecA superfamily

The results from the *recA* superfamily analyses indicated that there are indeed phylogenetically novel subfamilies of housekeeping genes in metagenomic data that have not yet been characterized. Is this finding unique to *recA*? To answer this, we selected another housekeeping gene for comparison: *rpoB*, the gene encoding the RNA polymerase β-subunit that carries out RNA chain initiation and elongation steps. *rpoB* is a universal gene found in all domains of life, as well as in many viruses. It has been adopted as a phylogenetic marker for studies of the Bacteria [57], the Archaea [58], and the Eukaryota [59], as well as for metagenomic studies of phylogenetic diversity in the Sargasso Sea [19]. Homologs of RpoB were identified in Genbank, genomes and the GOS metagenomic data using the same approach as for RecA with one significant difference. The RpoBs are large, multi-domain proteins, a large number of the *rpoB* sequences in the GOS data sets encode only partial peptides. Since this poses special complications for RpoB protein clustering, we excluded from our analysis RpoB peptides containing <400 amino acids.

In total, for further analysis we identified 1875 RpoB homologs from the GOS data set plus 784 known sequences from published microbial genomes [51] and the NRAA database. These known sequences included bacterial RpoBs as well as RNA polymerase subunit II proteins from the Eukaryota, the Archaea, and viruses. As with the RecA superfamily, RpoB clusters were identified using the Lek clustering algorithm (see Methods), here creating 17 such clusters that contain at least two members.

Nine of the 17 clusters contain only GOS sequences. Two of these (clusters 1 and 11) were determined to correspond to fragments of bacterial *rpoB*s and thus were excluded from further analysis. Four clusters (clusters 9, 10, 15, 16) correspond to peptides that only align to one end of known RNA polymerases and appear to be most closely related to eukaryotic RNA polymerases. These potentially could represent single exons of larger sequences and thus were excluded from further analysis. One cluster (cluster 5) contains only two sequences and though they appear to be full length, this family was excluded from further analysis because we chose to analyze only clusters with at least three sequences.

**Figure 3:**
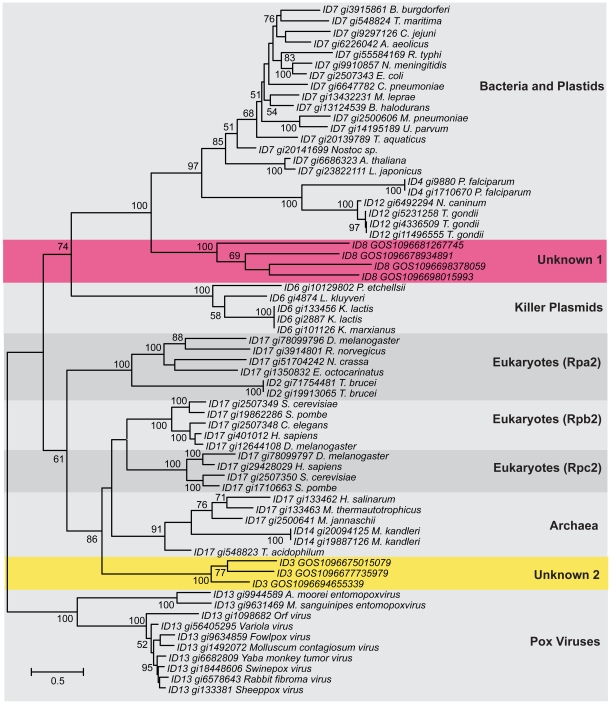
Phylogenetic tree of the RpoB superfamily. All RpoB sequences were grouped into clusters using the Lek algorithm. Representatives of each cluster that contained >2 members were then selected and aligned using MUSCLE. A phylogenetic tree was built by from this alignment using PHYML; bootstrap values are based on 100 replicas. The Lek cluster ID precedes each sequence accession ID. Proposed subfamilies in the RpoB superfamily are shaded and given a name on the right. The two novel RpoB clades that contain only GOS sequences are highlighted by the colored panels.

Representatives were then selected from the remaining clusters and used to build the RpoB superfamily tree (Figure 3). Based on the clusters and the tree structure, we divided the RpoB superfamily into the nine proposed *subfamilies* labeled in the tree. As with the RecA superfamily, there is a good correspondence between the Lek clusters and the tree suggesting that the Lek clustering did a reasonable job of identifying major RpoB groupings.

The largest number of homologs from the GOS data (1602 sequences) map to the Bacteria and Plastids RpoB clade, while the second largest number (181 sequences) group with the archaeal and eukaryotic clades. The relatedness of archaeal and eukaryotic RNA polymerases is consistent with previous observations [58]. Two other distinct clades on the tree correspond to RNA polymerases from yeast linear plasmids, including the toxin-producing killer plasmids [60], and the *Rpo2s* from viruses such as poxviruses [61].

Two of the RpoB subfamilies include only GOS sequences: **Unknown 2** which corresponds to Lek cluster 3 and **Unknown 1**, which corresponds to Lek cluster 8. These can be considered likely novel, previously unknown RpoB subfamilies. Both subfamilies are shown as deeply branching lineages in the phylogenetic tree (Figure 3) though we note the rooting of the tree is somewhat arbitrary. In terms of the organismal origin of the sequences in these subfamilies, we do not have a lot of information. The **Unknown 2** is peripherally related to the RpoB homolog from the giant Mimivirus (data not shown) and thus may represent uncharacterized relatives of mimivirus [80]. We have no useful information relating to the origin of the sequences in the **Unknown 1** subfamily.

That comparable results were obtained from both our *recA* and *rpoB* studies demonstrates the capability of our clustering and phylogenetic analysis methods to potentially identify deeply branching organisms from environmental metagenomic sequences.

### What do these novel groups represent?

The ultimate question concerning the novel subfamilies that we found is what is their origin? Lacking both visual observation and/or complete genomes, we do not currently have an answer. One trivial possibility is that they are artifacts of some kind (see [81] for a theoretical discussion of issues with artifacts in searching for phylogenetically novel organisms). In theory the novel sequences could represent chimeras, created in vitro from recombination between DNA pieces of different origins. We note that we focused our analysis on assembled contigs from the GOS data in a large part because annotation is more reliable for longer DNA segments. However, assembling metagenomic data has the potential to create artificial chimeras (much like in vitro recombination) and thus some assemblies may not represent real DNA sequences. We purposefully restricted our analysis to those subfamilies that have multiple members in order to avoid misleading results from rare chimeras or assembly artifacts; thus we think they likely represent real sequences.

Assuming the sequences are in fact real, we offer four possible biological explanations for their phylogenetic novelty. First, they could represent recombinants of some kind where domains from different known subfamilies have been mixed together to create a new form (e.g., perhaps the N-terminus of bacterial RecA was mixed with the C-terminus of a Rad51D). We consider this unlikely because the phylogenetic uniqueness for each group appears to be spread throughout the length of the proteins. A second possibility is that the novel sequences could represent paralogs resulting from ancient duplications within these gene families (and that these genes now reside in otherwise unexceptional, evolutionary lineages). We consider this extremely unlikely. Given the absence of representatives of these subfamilies from the sequenced genomes now available from dozens of the Eukaryota and Archaea and from hundreds of the Bacteria, this non-parsimonious explanation would require parallel gene loss of such ancient paralogs in most lineages in the tree of life, with gene retention in only a few organisms.

A third possibility is that the genes from novel subfamilies come from novel heretofore uncharacterized viruses. Given that the known viral world represents but a small fraction of the total extant diversity, and given some of the unexpected discoveries coming from viral genomics recently, this is entirely possible. For example, viruses have been characterized with markedly larger genomes that contain not only more genes, but genes previously found only in cellular organisms [62], [63]. In some cases, the viral forms of these genes appear to be phylogenetically novel compared to those in cellular organisms [62], [63].

It has not escaped our notice that the characteristics of these novel sequences are consistent with the possibility that they come from a new (i.e., fourth) major branch of cellular organisms on the tree of life. That is, their phylogenetic novelty could indicate phylogenetic novelty of the organisms from which they come. Clearly, confirmation or refutation of this possibility requires follow-up studies such as determining what is the source of these novel, deeply branching sequences (e.g., cellular organisms or viruses). Then, depending on the answers obtained, more targeted metagenomics or single-cell studies may help determine whether the novelty extends to all genes in the genome or is just seen for a few gene families.

Whatever the explanation for the novel sequences reported here, this discovery of new, deeply branching clades of housekeeping genes suggests that environmental metagenomics has the potential to provide striking insights into phylogenetic diversity, insights that complement those derived from rRNA studies. In the future we plan to explore more metagenomic data sets using an expanded collection of phylogenetic markers. Additional gene family classification and analysis tools, such as Markov clustering (MCL [64], [65]) and sequence similarity network visualization [64], [65], will further empower us to identify and understand these novel, deeply branching lineages—more of which may be waiting to be unveiled.

## Methods

### Identification of deeply-branching ss-rRNA sequences

A data set of 340 representative ss-rRNA sequences from all three domains was prepared. These sequences represented 134 eukaryotic, 186 bacterial, and 20 archaeal species. Alignments for these 340 sequences were extracted from the European Ribosomal RNA database [66] and then manually curated to remove columns with more than 90% gaps or with poor alignment quality. Sorcerer II Global Ocean Sampling Expedition (GOS) ss-rRNA sequences were identified by the PhylOTU pipeline [67]. Using MUSCLE [68], [69], each GOS ss-rRNA sequence was aligned with the representative alignments (using the representatives as a profile). A neighbor-joining tree including that sequence and the representative ss-rRNAs was then built using PHYLIP [70]. If a GOS sequence branched only one or two nodes away from the node separating the three domains, it was analyzed by the automated, phylogenetic tree-based ss-rRNA taxonomy and alignment pipeline (STAP) [39], [71], a protocol that draws upon the entire greengenes bacterial and archaeal ss-rRNA database [39], [71], as well as the SILVA database for eukaryotic ss-rRNAs [72].

### Identification of RecA and RpoB homologs in the GOS, microbial, and NRAA data sets

Homologs of RecA and RpoB were retrieved from the Genbank NRAA database (ftp://ftp.ncbi.nih.gov/blast/db/FASTA/nr.gz) and from all complete microbial genomes publicly available in November, 2009 [51]. Homologs of RecA and RpoB were defined by HMM profile screening of Pfam profiles (PF00154, PF08423, PF00562) [73], [74] and TIGRfam profiles (TIGR02012, TIGR02013, TIGR02236, TIGR02237, TIGR02239, TIGR03670) [75], as well as by BLASTP searches [76] using a diverse collection of known family members as query sequences. The retrieved sequences included representatives from the bacterial RecA, the archaeal RadA and RadB, the viral UvsX, and the eukaryotic DMC1, Rad51, Rad51B, Rad51C, and Rad51D families, among others. Likewise, RpoB homologs were identified, including the bacterial RpoB; the eukaryotic Rpa2, Rpb2, and Rpc2; and both archaeal and viral RNA polymerase subunit II. Known RecA and RpoB sequences were then used to query the GOS data set to identify homologs. For RpoB, only homologs containing >400 amino acids were included.

### Protein clustering

The 522 RecA homologs retrieved from the GenBank NRAA database (ftp://ftp.ncbi.nih.gov/blast/db/FASTA/nr.gz) and the published microbial genomes [51] were combined with 4125 RecA homologs retrieved from the GOS [35], [36] data set into one file. A Lek clustering algorithm was used to cluster the protein sequences into subfamilies [52] using a BLASTP E-value cutoff of 1e-40 and Lek clustering score cutoff of 0.10. A total of 40 clusters were generated, 23 of which have more than three members.

The same approach was used to cluster the 784 RpoB homologs from the NRAA database and published microbial genomes [51] and the 1875 RpoB homologs from the GOS data set. However, in this case, a BLASTP E-value cutoff of 1e-70 and Lek clustering score cutoff of 0.60 used. A total of 1816 GOS sequences and 778 RpoB homologs from NRAA and microbial genomes were clustered into 17 clusters containing more than two members. We note that for the novel RpoB clusters, confirmation that they were homologs of RNA polymerases was done by BLAST searches against Genbank and by HMM searches against the Pfam database of protein families.

For both the RecA and RpoB superfamily analysis, the cutoff values for the BLASTP search and the Lek clustering were chosen such that the clusters produced were reasonably comparable to the annotation of the sequences (e.g., RecAs in one cluster, Rad51 in another).

### Phylogenetic tree building

Representative amino acid sequences from each of the RecA and RpoB clusters were selected manually and then aligned by MUSCLE [68]. The alignments were examined and manually trimmed to ensure alignment quality. A maximum likelihood tree was built from the curated alignments using PHYML [77]. For phylogenetic tree construction, bootstrap values were based on 100 replicas, the JTT substitution model was applied [78], and both the proportion of invariable sites and the gamma distribution parameter were estimated by PHYML.

### Analysis of assemblies containing novel RecA sequences

Five RecA subfamilies (corresponding to sequences in clusters 2, 5, 9, 11, and15) contain only GOS sequences (i.e., they were novel metagenomic only subfamilies) and also contain complete genes (i.e., they were not made up of only sequence fragments). In total, these clusters contain 24 metagenomic RecA homologs. We examined the 24 GOS assemblies that encode these RecA homologs. From these we retrieved 559 putative protein-encoding genes. Of these 24 assemblies, 12 contained a combined total of 55 genes with BLASTP hits in the NRAA database (E-value cutoff of 1e-5). We assigned gene functions to the 55 genes based on their top BLASTP hits. For each of these 55 genes, a phylogenetic tree was built by QuickTree [79] using the amino acid sequences of their top 50 BLASTP hits in the NRAA database. A putative “taxonomy” at the domain level was assigned based on their nearest neighbor in the phylogenetic tree.

Assembly 1096627390330, the largest of the 12 assemblies, was analyzed further. Translation in all six frames yielded 114 potential ORFs. Functions could be assigned to 33 of the 114 based on similarity to genes in the NRAA database using BLASTP. A gene map (Figure 2) was built of the entire assembly including the 33 annotated genes plus 16 hypothetical proteins, i.e., ORFs without annotation that do not overlap any of the 33 genes. When non-annotated ORFs overlapped, the longest ORF was used to represent the group on the map.

### Data and protocol availability

We've made the following data and protocols available for the public: (1) GOS and reference sequences for RecA and RpoB; (2) Subfamilies of RecA and RpoB (Table 1,3); (3) Alignments and Newick format phylogenetic trees of RecA and RpoB (Figure 1,3); (4) Sequences of the genes that share assemblies with the novel *recAs.* (Table 2); (5) GOS ss-rRNA sequence reads; (6) the Lek clustering program. The data and protocols are available at http://bobcat.genomecenter.ucdavis.edu/GOSrecA_DATA/index.html. The data have also been submitted to the Dryad repository http://datadryad.org/ - http://dx.doi.org/10.5061/dryad.8384.

**Table 3:** RpoB subfamilies.

Cluster ID	Corresponding Subfamily (see Figure 3)	Comments	GOS Only?	Number of GOS Sequences
7	Bacteria and Plastids			1602
4	Bacteria and Plastids			0
12	Bacteria and Plastids			0
8	Unknown 1		+	4
6	Killer Plasmids”			0
17	Rpa2/Rpb2/Rpc2/Archaea	Includes most eukaryotic (nuclear) and archaeal superfamily members		181
2	Rpa2			0
14	Archaea			0
3	Unknown 2		+	3
13	Pox Viruses			0
*1	n/a	Partial sequences likely from bacteria	+	6
*11	n/a	Partial sequences likely from bacteria	+	2
*9	n/a	Partial sequences likely from eukaryotes.	+	4
*10	n/a	Partial sequences likely from eukaryotes.	+	4
*15	n/a	Partial sequences likely from eukaryotes.	+	3
*16	n/a	Partial sequences likely from eukaryotes.	+	5
**5	n/a	Not analyzed further because only two representatives identified	+	2

A Lek clustering method was applied to all RpoB superfamily members retrieved from the NRAA database, microbial genome projects, and the GOS data set. Clusters that contain only sequences from the GOS data set are noted as “From GOS only.”

*Clusters 1, 9, 10, 11, 15, and 16 contain only sequence fragments from the GOS data set; though possibly novel they were omitted from further analysis.

**Cluster 5 contains only two sequences. Though both are from the GOS (IDs 1096695464231 and 1096681823525) and may represent a novel RpoB subfamily, this group was excluded from further analysis because we restricted analyses to groups with three or more sequences.
